# Risk and Protective Factors for Suicide in Patients with Alcoholism

**DOI:** 10.1100/tsw.2006.254

**Published:** 2006-10-31

**Authors:** Leo Sher

**Affiliations:** Department of Psychiatry, Columbia University New York State Psychiatric Institute, New York, NY, United States

**Keywords:** alcoholism, suicide, depression, risk, protective factors, United States

## Abstract

Alcoholism is associated with a high risk for suicidal behavior. Up to 40% of persons with alcoholism attempt suicide at some time and 7% end their lives by committing suicide. Risk factors include being male, older than 50 years of age, living alone, being unemployed, poor social support, interpersonal losses, continued drinking, consumption of a greater amount of alcohol when drinking, a recent alcohol binge, previous alcohol treatment, a family history of alcoholism, a history of comorbid substance abuse (especially cocaine), a major depressive episode, serious medical illness, suicidal communication, and prior suicidal behavior. Suicidal behavior is especially frequent in patients with comorbid alcoholism and major depression. However, all patients with alcoholism should be evaluated for suicide risk. Understanding of risk and vulnerability to suicidal behavior in alcoholism still outweighs our knowledge of protective factors and resilience. Knowledge of protective factors for suicide may help to prevent and/or predict suicidal behavior. Protective factors for suicide in alcoholism are quite varied and include an individual's biological and behavioral characteristics, as well as attributes of the environment and culture. Protective factors include effective clinical care for psychiatric (including alcoholism and drug abuse) and physical disorders, easy access to a variety of clinical interventions and support for seeking help, restricted access to highly lethal means of suicide, strong connections to family and community support, skills in problem solving and conflict resolution, cultural and religious beliefs that discourage suicide and support self-preservation. Future studies are necessary to determine which interventions may reduce suicidal behavior in alcoholism.

### INTRODUCTION

In most Western societies, at least 90% of people consume alcohol at some time during their lives, and 30% or more of drinkers develop alcohol-related life problems[1]. Severe alcohol-related life impairment, alcohol dependence (alcoholism), is observed at some time during their lives in 10% of men and 3–5% of women, with an additional 5–10% of each sex developing persistent, but less-intense problems that are diagnosed as abuse. There is no scientifically acceptable explanation why some develop problems and most do not. Chronic excessive use of alcohol produces a wide range of psychiatric symptoms that in various combinations can mimic other psychiatric disorders. Therefore, while a person is drinking heavily and during the withdrawal period, it is difficult to determine whether he or she is suffering from a psychiatric condition other than alcoholism.

Alcoholism is associated with a high risk for suicidal behavior[2,3,4]. Up to 40% of persons with alcoholism attempt suicide at some time, and up to 7% end their lives by committing suicide. Understanding of risk and vulnerability to suicidal behavior in alcoholism still outweighs our knowledge of protective factors and resilience. Knowledge of protective factors for suicide may help to prevent and/or predict suicidal behavior. Risk and protective factors are interrelated.

### STRESS-DIATHESIS MODEL OF SUICIDAL BEHAVIOR

Most patients with psychiatric disorders do not commit suicide, indicating that other factors influence risk. Suicide attempters have a tendency to make more than one suicide attempt, sometimes with increasing lethality in succeeding attempts[5]. Malone et al.[5] reported that vulnerable individuals with a mood disorder who make a suicide attempt do so early in the course of illness.

Risk factors may be thought of as leading to or being associated with suicide; that is, people "possessing" the risk factor are at greater potential for suicidal behavior[6,7,8]. Protective factors, on the other hand, reduce the likelihood of suicide. They enhance resilience and may serve to counterbalance risk factors. Risk and protective factors may be biopsychosocial, environmental, or sociocultural in nature. Although this division is somewhat arbitrary, it provides the opportunity to consider these factors from different perspectives. Understanding the interactive relationship between risk and protective factors in suicidal behavior and how this interaction can be modified are challenges to suicide prevention. Unfortunately, the scientific studies that demonstrate the suicide prevention effect of altering specific risk or protective factors remain limited in number. However, the impact of some risk factors can clearly be reduced by certain interventions, such as providing lithium for bipolar disorder or strengthening social support in a community[8,9,10].

Suicide is not simply a logical response to extreme stress and this conclusion led to the hypothesis of a stress diathesis model of suicidal behavior[7,8]. The diathesis or predisposition to suicidal behavior is a key element that differentiates psychiatric patients who are at high risk from those at lower risk. The objective severity of the psychiatric illness or number of recent life events do not assist in identifying patients at high risk for suicide attempt. Instead, it is the impact of the illness or life events on the person that varies greatly and is correlated with suicidal behavior.

Risk and protective factors determine how an individual reacts to a given stressor and depends on factors that mold personality, such as environmental and genetic factors, childhood experiences, etc. Risk and protective factors to suicidal behavior are influenced by genetic factors, parenting, psychiatric and medical illnesses (especially affecting the brain, e.g., epilepsy, migraine, Huntington’s disease, alcoholism and substance abuse), and cholesterol level[7,8,11,12,13,14,15,16,17,18,19]. Some of these factors may be interrelated.

Examples of stressors associated with suicidal acts include the onset or worsening of an episode of major depression, use of alcohol or sedatives that may disinhibit patients, an acute medical illness especially affecting the CNS, and finally, adverse life events[7,8,16,17,18,19]. Stressor is a precipitant that determines “timing” of a suicide attempt.

A model of suicidal behavior among individuals with alcoholism has recently been proposed[17]. Predisposing factors that are presumed to increase (moderate) risk for suicide among individuals with alcoholism are aggression/impulsivity and alcoholism severity, which represent predominantly externalizing constructs, and negative affect and hopelessness, which represent predominantly internalizing constructs. Major depressive episodes and stressful life events, particularly interpersonal difficulties, are conceptualized as precipitating factors. This model is consistent with the stress-diathesis model of suicidal behavior[7,8]. Interpersonal events, especially partner-relationship disruptions, are most strongly associated with attempted and completed suicide in persons with alcoholism[17,19]. Disruptions in interpersonal relationships occur more frequently before suicide among individuals with alcohol and other substance use disorders than in patients with other psychiatric disorders. Partner-relationship disruptions related to discord, such as divorce, separation, and break-up of steady dating, are most common. Elevated aggression and impulsivity may promote interpersonal disruptions that, in turn, increase risk for a suicidal behavior.

### DEMOGRAPHIC CHARACTERISTICS AND SUICIDAL BEHAVIOR

#### Gender

In the general population, the rate of suicide is about four times higher in males compared with females, and yet females attempt suicide three times more likely than males[8,16]. Part of the reason for the higher suicide rate in men is accounted for by the more violent means of attempt used by men. Qin et al.[20] suggested that gender differences could not be explained by differential exposure to known risk factors. In some countries, such as China, the suicide rate in women is similar to that of men, perhaps due to use of highly lethal methods by women[8].

Generally, analysts are divided over how to explain the elevated risk of suicide for older white men[21]. Some researchers point to a lack of resilience or coping ability. Others point to men's choice of more lethal means of suicide. More generally, systemic obstacles related to the primary care system (as well as cultural bias that assumes depression is a natural feature of aging) also inhibit detection of older people at risk of suicide. Some researchers argue that older white males lack the resilience and coping mechanisms that make older white women and older black people less prone to suicide[21]. The lower suicide rates among women suggest that women are capable of more complex and flexible coping strategies than men. The higher suicide rate in men may be related to higher consumption of alcohol.

#### Age

Suicide rates in females are generally stable with age after the midteens until the very oldest age groups (85+), where there usually is a slight drop off[8]. Elderly white males have a big increase in suicide rates beginning in their 70s. In the U.S., the adolescent suicide rate approximately tripled from 1950 until 1980, but has begun to fall. Suicide rates in the entire population have fallen about 9% from 1988 to 1998. Generally, among males, being younger is a protective factor against suicide.

A recent study has found that age may also play a role in the risk for suicide among patients with alcoholism[22]. The authors found that middle-aged and older patients with alcoholism are at a greater risk for suicide than younger patients. The researchers looked at data collected in New Zealand on 193 adults who had committed suicide and on 240 adults who made a serious suicide attempt that required at least 24 h of hospitalization. The researchers found that while the association between alcohol use and completed suicide increased with age, the association between alcohol use and suicide attempts was not affected by age. Furthermore, increased age amplified the association between mood disorders and suicide, while decreased age strengthened the association between mood disorders and suicide attempts. Several hypotheses for these findings were given: (1) older alcoholics have been exposed to the deleterious effects of alcohol for a longer period of time; (2) aggression and impulsivity underlie the failure to mature out of alcoholism and the risk for suicide; (3) older alcoholics at risk for suicide have a later onset of alcoholism in the context of a negative affect; and (4) older alcoholics have diminished physical reserves, which makes surviving a suicide attempt less likely.

### Race

About 90% of all suicides in the U.S. are committed by whites[8,16]. Most studies demonstrate that Caucasians are at highest risk for suicide, followed by Native Americans, African Americans, Hispanic Americans, and Asian Americans. For example, in community sample studies, Anglo bipolar patients have higher rates of suicide attempt (32% are attempters vs. 68% nonattempters), than African Americans (21% are attempters) or Hispanic Americans (13% are attempters)[23]. The reasons for ethnic differences in suicide rates are unknown.

### Marital Status

In general, people who are unmarried, divorced, or widowed are at higher risk of suicide[8,16]. Highest to lowest rates in the U.S. are found in the following order: widowed, divorced, single or never married, married, and married with children. The protective effect of marriage against suicide is especially marked among males and whites. Studies suggest that having children may serve as a protective factor against suicide in patients with mood disorders and in the general population[24,25].

### Education and Social Status

Most studies that have examined associations between measures of social disadvantage and suicide or suicide attempt have reported increased risk of suicidal behavior among individuals from socially disadvantaged backgrounds characterized by such features as low socioeconomic status, limited educational achievement, low income, and poverty[26,27,28]. For example, odds of suicide were found to be twice as high among individuals of lower socioeconomic status compared to those from more advantaged backgrounds[26], and risk of suicide was shown to be elevated among young people who had dropped out of school or were not at college[27]. Higher socioeconomic status protects against suicide.

### Religion

Suicide rates are lower in religious countries than in secular ones[29,30]. Some of this difference may be due to underreporting in religious countries because of concerns over stigma. Yet, some of the difference may be real, although it is not known whether the negative association between religion and suicide is due to its integrative benefits (such as social cohesion, as proposed by Durkheim[31]) or to the moral imperatives of religious belief, given its prohibitions against suicidal behavior[29,30,32]. Most previous studies have been epidemiologic and have investigated the association between completed suicide and religion. An inverse relationship between religious commitment and suicidal ideation has also been reported[30,32].

## CLINICAL CHARACTERISTICS AND SUICIDAL BEHAVIOR

### Role of Psychiatric and Medical Disorders

Conditions associated with a high risk for attempted suicide and completed suicide include alcohol and substance abuse, mood disorders, and Cluster B personality disorders[8,17,18,19]. In their follow-up study, Angst and Preisig[33] found no difference in rates of suicide in patients with unipolar depression compared to patients with bipolar disorder. There is disagreement in the literature regarding the role of relationship of mood disorder subtype to suicide[8,34,35,36]. Unlike Angst and Preisig[33], most studies do not involve long-term follow-up, but are psychological autopsies of completed suicides or short-term follow-up studies. For example, Rihmer et al.[34] studied 100 consecutive suicide victims with major depression and found that 46% of them had bipolar II disorder. Bulik et al.[35], studying recurrent major depression in patients without Axis II pathology, noted that the presence of alcohol abuse was significantly more common in suicide attempters. The association between comorbid substance abuse and suicide attempts was found in both genders and in all age groups. Research in general psychiatric populations suggest that alcoholism, substance abuse, and smoking may share a common predisposition with the risk for suicide, or may directly contribute to suicide risk through facilitating disinhibition, depressed mood, or other neurochemical effects on the brain[18,19,37].

Physical illness increases the risk for suicide and suicidal behavior[7,8,10,12,13,16]. Certain illnesses that affect the brain such as AIDS, epilepsy, Huntington's disease, stroke, head injury, or chronic renal failure confer greater risk of suicide than others. Medical disorders are common among patients with alcoholism and may contribute significantly to suicide risk.

#### Presence of Specific Symptoms and Personality Traits

Presence of specific symptoms may play a role in suicidal behavior. As a result of the National Institute of Mental Health Collaborative Depression study, three group of symptoms: (1) anhedonia, hopelessssness; (2) anxiety, agitation, panic; (3) aggression, impulsivity; have been put forward as more predictive of suicide than either diagnoses or syndromes[38]. Elevated aggression and impulsivity are common among patients with alcoholism. Beck[39] suggests that cognitive dysfunctions (particularly hopelessness) are a strong predictor of suicidal behavior in persons with affective disorders.

The role of psychotic symptoms in suicidal behavior in persons with mood disorders remains controversial[8]. Several studies found no support for increased suicidality in persons with psychosis and affective disorder[8]. However, some studies support the association of the symptom of delusional thinking or other psychotic features in major depression and suicide[8,40,41].

#### Childhood Stressors

A family history of parental separation or divorce is associated with increased risks of suicide or attempted suicide[28,42,43]. Suicide victims, for example, have been found to be more likely than control subjects to come from a "nonintact family of origin", even after controlling for demographic factors[43]. Adolescents who made suicide attempts were reported to be more likely to have experienced at least three changes of parental figures between age 5 years and 15 years compared with those not making attempts[42]. Positive childhood experiences may increase resilience to suicidal behavior in adulthood.

### CONCLUSIONS

Risk factors for attempted and completed suicide in individuals with alcoholism include being male, older than approximately 50 years of age, living alone, being unemployed, poor social support, interpersonal losses, continued drinking, consumption of a greater amount of alcohol when drinking, a recent alcohol binge, previous alcohol treatment, a family history of alcoholism, a history of substance abuse (especially cocaine), major depressive episode, serious medical illness, suicidal communication, and prior suicidal behavior. Suicidal behavior is especially frequent in patients with comorbid alcoholism and major depression. However, all patients with alcoholism should be evaluated for suicide risk. Protective factors for suicide in alcoholism are quite varied and include an individual's biological and behavioral characteristics, as well as attributes of the environment and culture. Protective factors include effective clinical care for psychiatric (including alcoholism and drug abuse) and physical disorders, easy access to a variety of clinical interventions and support for seeking help, restricted access to highly lethal means of suicide, strong connections to family and community support, skills in problem solving and conflict resolution, cultural and religious beliefs that discourage suicide and support self-preservation[44]. To prevent suicidal behavior, we need to develop new approaches to suicide prevention by enhancing our understanding of preventive interventions grounded in knowledge of risk and protective factors.
